# Identifying optimal ranges of weight gain at the end of the second trimester result from a population-based cohort study

**DOI:** 10.1017/S1368980023001490

**Published:** 2023-10

**Authors:** Shuang Zhang, Nan Li, Wei Dong, Weiqin Li, Guangyan Cheng, Hong Zhu, Wen Yang, Baocheng Chang, Junhong Leng

**Affiliations:** 1 Tianjin Women’s and Children’s Health Center, Tianjin, People’s Republic of China; 2 NHC Key Laboratory of Hormones and Development, Tianjin Key Laboratory of Metabolic Diseases, Chu Hsien-I Memorial Hospital & Tianjin Institute of Endocrinology, Tianjin Medical University, Tianjin, People’s Republic of China; 3 Tianjin Central Hospital of Gynecology Obstetrics, Tianjin, People’s Republic of China; 4 Department of Epidemiology and Biostatistics, School of Public Health, Tianjin Key Laboratory of Environment, Nutrition and Public Health, Tianjin Medical University, Tianjin, People’s Republic of China; 5 Nan Kai District Center for Disease Control and Prevention, Tianjin, People’s Republic of China

**Keywords:** Adverse maternal and infant outcomes, Cohort study, Gestational weight gain, Optimal range

## Abstract

**Objective:**

To identify the optimal weight gain at the end of the second trimester.

**Design:**

This was a population-based cohort study from the antenatal care system in Tianjin, China. We calculated gestational weight gain (GWG) based on the weight measured in the first trimester and the end of the second trimester. Restricted cubic spline analysis was performed to model the possible non-linear relationships between GWG and adverse outcomes. The optimal GWG was defined as the value of the lowest risk. Non-inferiority margins and the shape of the spline curves identified the recommended ranges in Chinese-specific BMI categories.

**Setting:**

Tianjin Maternal and Child Health Cohort.

**Participants:**

Singleton pregnant women aged 18–45 years.

**Results:**

In total, 69 859 pregnant women were included. Adverse outcome (including stillbirth, preterm birth, hypertensive disorders of pregnancy, gestational diabetes mellitus, small and large for gestational age) was significantly associated with GWG at the end of the second trimester. The risk score was non-linearly correlated with GWG in the underweight, normal weight and overweight groups. GWG at the end of the second trimester should not be < 7 kg in underweight group. For most normal-weight women, a GWG of about 8 kg is optimal. Pregnant women who are overweight should not have a GWG of more than 9 kg. We advised women with overweight and obesity to keep positive growth of GWG (> 0 kg) in the first and second trimesters.

**Conclusions:**

According to the comprehensive adverse maternal and infant outcomes, we recommend the optimal GWG at the end of the second trimester. This study may provide a considerable reference for weight management.

Gestational weight gain (GWG) reflects maternal nutrition status and the intrauterine nutritional environment of offspring before birth^(1)^. It is closely associated with the risk of metabolic disorders and other health outcomes of mothers and children^(2–4)^. Insufficient GWG was associated with a higher risk of small for gestational age (SGA), low birthweight and preterm birth. Excessive GWG was associated with a higher risk of large for gestational age (LGA), macrosomia and cesarean delivery^(5)^. The Institute of Medicine (IOM) has recommended the ranges of GWG at term and the weekly weight gain in the second and third trimesters^(6)^. GWG assessment has become a widely implemented component of prenatal care in most countries and regions. A meta-analysis on GWG across continents and ethnicity^(7)^ showed that women in the United States and Europe had higher prepregnancy BMI. They also had a higher prevalence of GWG above guidelines and a lower rate of GWG below the guideline than women in Asia. However, when applying Asian regional BMI categories, rates of GWG above guidelines and related clinical outcomes were similar across continents. With the development of prenatal care, weight assessment and intervention were becoming more accurate^(8)^. Some studies have established specific GWG recommendation ranges for local populations^(9)^, while others have focused on the effects of GWG in different trimesters on pregnancy outcomes^(10)^.

Despite the ample evidence from previous studies on full-term GWG, obstetric and prenatal care physicians are eager to start weight management earlier. Weight assessment in the first trimester has been hindered in practice. Limited by the local economic and medical level, coupled with someone’s poor health awareness, many women do not start antenatal examinations until the second trimester. In addition, maternal weight fluctuates due to physical conditions such as hyperemesis gravida in the first few weeks of pregnancy. These all inhibit the feasibility of weight assessment in the first trimester. Instead, weight assessment during the second trimester was a better candidate: (1) the initial symptoms of nausea, vomiting and other symptoms of discomfort have been relieved, diet and physical activity have been improved and the pregnant woman’s weight steadily and rapidly increased. (2) In most countries and regions, gestational diabetes screening occurs between 24 and 28 weeks of pregnancy. It is very convenient for pregnant women to measure their weight at this time. It can evaluate the nutritional status in the first and second trimesters and be an essential basis for guiding lifestyle intervention in the third trimester. However, there is yet to be a unified standard for evaluating GWG at the end of the second trimester.

Based on a full-fledged antenatal care system subordinate to the government, this study established a mother–infant cohort covering the community population in Tianjin, China. We attempted to establish recommended ranges of GWG at the end of the second trimester.

## Methods

### Study population and data collection

This study included pregnant women who received prenatal care in the Tianjin Women and Children Health Care System. The system covers all communities in Tianjin, and the antenatal care coverage rates of the local pregnant population exceed 95 %. We obtained medical records of pregnancy, including essential characteristics (e.g. age, ethnicity and education), family history of diseases, history of diseases (e.g. diabetes and hypertension), lifestyle habits (e.g. smoking) and menstrual and obstetrical history at the first prenatal visit. The results, including height, weight, blood pressure measurements and complications during pregnancy (e.g. gestational diabetes mellitus (GDM) and hypertensive disorders of pregnancy (HDP)), were also recorded continuously until the end of pregnancy (abortion, induce of labour or delivery). Birth information was also documented (e.g. gestational age at birth, birth length and weight and Apgar score). All data were recorded and checked by obstetricians and nurses, and strict quality control was routinely performed. Pregnant women were included in this cohort if they met the following criteria: singleton pregnancy; aged 18–45 years; complete follow-up records from the initial prenatal examination (not later than 13 weeks + 6 d of gestation) to the end of pregnancy (not earlier than 24 weeks + 0 d of gestation) and maternal weight during 24–28 weeks of gestation was necessary.

### Gestational weight gain and BMI

In this study, baseline weight was defined as maternal weight measured at the first antenatal visit to a community hospital^(8,11)^. The maternal weight at the end of the second trimester was measured during 24–28 weeks of gestation. Mean weight and gestational age were calculated if maternal weight was measured more than or equal to twice during this period. We calculated GWG as the difference between the weight at the end of the second trimester and the baseline weight. Gestational age was determined based on the last menstrual period, which was adjusted using first-trimester ultrasound. GWG less than the lower cut-off value of the recommended range was evaluated as insufficient, and more than the upper cut-off was considered excessive. Prepregnancy BMI was calculated as the baseline weight in kilograms divided by height in meters squared. BMI categories followed the Chinese standard: underweight, < 18·5 kg/m^2^; normal weight, 18·5–23·9 kg/m^2^; overweight, 24·0–27·9 kg/m^2^ and obesity, ≥ 28·0 kg/m^2(12)^.

### Composite adverse outcomes and risk score

We defined the *composite adverse outcome* as the presence of one or more events: stillbirth, preterm birth, HDP, GDM, SGA and LGA. Stillbirth was defined as fetal death, intrapartum stillbirth or neonatal death in the first seven days of life. Preterm birth was defined as birth before 37 weeks gestation. HDP includes pregnancy-induced hypertension, preeclampsia, eclampsia, chronic hypertension with pregnancy and chronic hypertension with preeclampsia. GDM was diagnosed by a plasma glucose level greater than or equal to 5·1, 10·0 or 8·5 mmol/l at 0, 1 or 2 h in a 75 g oral glucose tolerance test^(4)^. SGA or LGA was defined as birth weight below the gestational week and sex-specific 10th percentile or above the 90th percentile of the Chinese reference values^(13)^.

Above, we compound six adverse events with equal weight as a single outcome. It was worth noting that some outcomes were more severe than others (e.g. a stillbirth is more severe than GDM). Recent research rated the seriousness of maternal and infant health outcomes linked with GWG^(14)^. Based on this, we tried to predict the optimal GWG ranges with risk score as the weighted outcome variable. The risk score of adverse outcomes was calculated by weighting the severity of the maternal and child health outcomes^(14)^, and the equation is as follows:

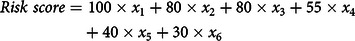




In the formula, *x*_1_, *x*_2_, *x*_3_, *x*_4_, *x*_5_ and *x*_6_ correspond to stillbirth, preterm birth, HDP, GDM, SGA and LGA, respectively. If an individual adverse event occurs, *x* is assigned a value of 1; if this event does not happen, *x* is assigned to 0. When a woman was diagnosed with GDM and gave birth to LGA during this pregnancy, her risk score of adverse outcomes was 85. The higher the risk score, the more severe the health outcome.

### Statistical analyses

All analyses were performed using IBM SPSS Statistics for Windows (Version 21.0. IBM Corp) and R software (Version 4.2.1, Project Comprehensive R Archive Network). Normally distributed continuous variables were presented as mean (sd), and categorical variables were expressed as numbers (percentages). We used restricted cubic splines (RCS) to flexibly model and visualise the relationship between GWG and adverse outcomes (apply the ‘rcssci’ package in R software). Maternal age, maternal height, gestational age of weight measure, parity (primipara or multipara), history of stillbirth (no verse yes), education (≤ 12 years or > 2 years) and active smoking (no verse yes) were adjusted as confounding factors. The numbers and positions of knots were assessed to identify the best-fitting model using the Akaike Information Criterion criteria. Two-tailed *P* < 0·05 was considered statistically significant.

### Establish the ranges of recommended weight gain

#### Non-inferiority margins

We use the non-inferiority margins as a quantitative approach for establishing the optimal range of GWG. Non-inferiority trials are a type of randomised clinical trial to determine whether a new intervention is at least as good as (not meaningfully worse than) a standard intervention^(15–17)^. The width of margins (e.g. 10 %, 15 % and 20 %) would depend on the prevalence of the adverse outcome in the control group^(17)^. For example, a hypothetical non-inferiority trial sets a prespecified non-inferiority margin of 10 % (RR: 1·10). If the upper limit of the 95 % CI (1·09) was just below the margin of 1·10, it could be concluded that the new intervention was non-inferior to the standard intervention^(18)^. Non-inferiority margins can also be used with observational data to identify points on the continuous increase of GWG where risks become meaningfully increased compared with the referent value (e.g. the GWG at which the risk of adverse outcomes was the lowest)^(18)^. The approach ensures that the cut-offs for recommended ranges reflect weight gains above or below which risks are deemed unacceptably increased. Furthermore, the margin can be expressed in either absolute terms (e.g. a risk difference of no more than 5 per 100) or relative terms (e.g. an increase in the risk of no more than 15 %)^(18)^.

#### Cut-off for *composite adverse outcome*

First, possible non-linear relationships between GWG and composite adverse outcomes were examined with RCS. The Y-axis represented the odds ratios for *the composite adverse outcome* (binary variable), and the X-axis represented GWG (continuous variable). The GWG value at OR = 1 was taken as the reference value. The concept of the non-inferiority test was simulated to delineate the non-inferiority margins at 10 %^(17)^. The cut-off values were selected according to the non-inferiority margins.

#### Cut-off for risk score of adverse outcomes

Second, we fitted the RCS curse that the Y-axis represents the risk score (continuous variable), and the X-axis is GWG (continuous variable). We took the GWG value at the lowest point of the curve (the nadir of risk) as the optimal GWG. This weight gain value served as the referent, and the cut-off value was selected according to the shape of the RCS curve^(19)^. The cut-off values coming from these curves were presented as candidates.

## Results

### Basic characteristics

Overall, 76 514 pregnant women (aged 18–45 years) accessed antenatal care (gestational age at first check < 14 weeks) in the Tianjin Women and Children Health Care System from 1 January 2015 to 31 December 2015. We excluded the women who were multiple pregnancies (*n* 1106) or diagnosed with diabetes or hypertension before the current pregnancy (*n* 257) or terminated their pregnancies earlier than 24 weeks gestation (*n* 3193). We enrolled 71 958 women in the cohort and recorded their weight at 24–28 weeks. We followed them closely until the end of the pregnancy. Of those, 290 were stillbirths, 3079 were preterm births, 2082 were HDP, 7198 were GDM, 3630 were SGA and 7884 were LGA. A total of 2099 pregnant women were not followed up because they transferred to other provinces for delivery. Finally, we analysed 69 859 pregnant women with completed follow-ups (online Supplementary eFig. 1).

Seven-thousand one-hundred twenty-three women (10·2 %) were categorised in the underweight group, 41 629 (59·6 %) in the normal weight group, 14 621 (20·9 %) in the overweight group and 6486 (9·3 %) in the obesity group. Their basic characteristics are presented in the population and four prepregnancy BMI groups (Table 1). As prepregnancy BMI increased, the proportion of lower education level (≤ 12 years), multipara, habitual smoking, first-degree relatives having hypertension or diabetes, history of stillbirth and cesarean delivery tended to increase. The incidence of composite adverse outcomes was 21·4 %, 26·0 %, 38·1 % and 50·1 % in the underweight, normal weight, overweight and obesity groups.

**Table 1:** General clinical characteristics of pregnant women in the cohort

Characteristic			Prepregnancy BMI groups*							
	All women		Underweight		Normal weight		Overweight		Obesity	
	*n*	%	*n*	%	*n*	%	*n*	%	*n*	%
*n* (%)	69 859	100	7123	10·2	41 629	59·6	14 621	20·9	6486	9·3
Maternal age, year										
Mean	28·17		26·70		28·08		28·89		28·72	
sd	4·14		3·61		4·02		4·37		4·44	
Maternal height, cm										
Mean	162·68		162·78		162·62		162·68		162·93	
sd	4·83		4·76		4·76		4·86		5·29	
Ethnicity										
Han	66 883	95·7	6838	96·0	39 823	95·7	14 008	95·8	6214	95·8
Minorities	2976	4·3	285	4·0	1806	4·3	613	4·2	272	4·2
Education										
≤ 12 years	26 117	37·4	2516	35·3	14 057	33·8	6075	41·5	3469	53·5
> 12 years	43 742	62·6	4607	64·7	27 572	66·2	8546	58·5	3017	46·5
Parity										
Primipara	49 001	70·1	5864	82·3	30 167	72·5	9114	62·3	3856	59·5
Multipara	20 858	29·9	1259	17·7	11 462	27·5	5507	37·7	2630	40·5
Active smoking	278	0·4	33	0·5	141	0·3	63	0·4	41	0·6
First-degree relatives have hypertension	3099	4·4	227	3·2	1732	4·2	760	5·2	380	5·9
First-degree relatives have diabetes	1546	2·2	126	1·8	790	1·9	380	2·6	250	3·9
History of stillbirth	365	0·5	19	0·3	189	0·5	119	0·8	38	0·6
Gestational age at delivery, week										
Mean	39·46		39·41		39·26		39·00		39·35	
sd	1·36		1·42		1·54		1·75		1·48	
Cesarean delivery	35 717	51·1	2607	36·6	19 447	46·7	8995	61·5	4668	72·0
Fetal sex†										
Male	35 990	51·5	3577	50·2	21 514	51·7	7579	51·9	3320	51·2
Female	33 861	48·5	3546	49·8	20 112	48·3	7038	48·1	3165	48·8
Incidence of adverse outcome										
*Composite adverse outcome*										
Mean	21 176		1521		10 834		5572		3249	
sd	30·3		21·4		26·0		38·1		50·1	
Stillbirth	290	0·4	18	0·3	169	0·4	63	0·4	40	0·6
Preterm birth	3078	4·4	246	3·5	1579	3·8	752	5·1	501	7·7
HDP	2082	3·0	68	1·0	696	1·7	628	4·3	690	10·6
GDM	7198	10·3	325	4·6	3370	8·1	2087	14·3	1416	21·8
SGA	3630	5·2	646	9·1	2196	5·3	562	3·8	226	3·5
LGA	8774	12·6	349	4·9	4245	10·2	2691	18·4	1489	23·0

BMI, body mass index; HDP, hypertensive disorders of pregnancy; GDM, gestational diabetes mellitus; SGA, small for gestational age; LGA, large for gestational age.

Values are mean (sd) or *n* (%).

* Prepregnancy BMI groups were divided following the Chinese standard: underweight, < 18·5 kg/m^2^; normal weight, 18·5–23·9 kg/m^2^; overweight, 24·0–27·9 kg/m^2^; obesity, ≥ 28·0 kg/m^2^.

† Sex of the eight fetuses (stillbirth) was not recorded.

We constructed GWG at 25·96 (sd 0·94) weeks. GWG decreased with the increase in prepregnancy BMI. In the underweight, normal weight, overweight and obesity groups, the median (P25–P75) of GWG was 8·6 (6·7–10·6) kg, 8·0 (6·0–10·1) kg, 7·0 (4·8–9·4) kg and 5·5 (3·0–7·9) kg, respectively. In the following analysis, we report the results of different BMI subgroups.

### Gestational weight gain and composite adverse outcomes

**Fig. 1:**
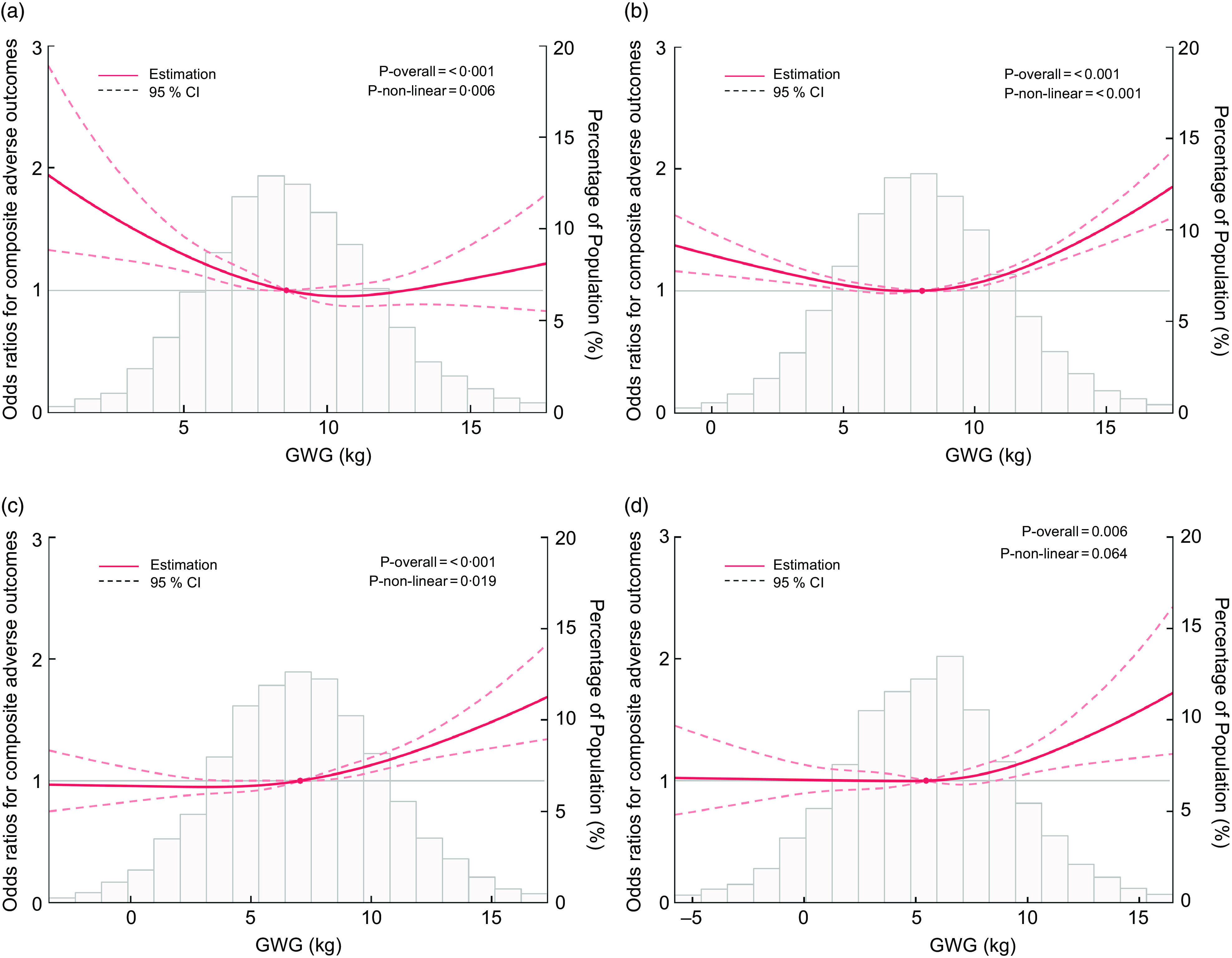
Restricted cubic spline for the associations between weight gain and composite adverse outcomes. (a) underweight group, (b) normal weight group, (c) overweight group, (d) obesity group. Prepregnancy BMI groups were divided following the Chinese standard: underweight, < 18·5 kg/m^2^; normal weight, 18·5–23·9 kg/m^2^; overweight, 24·0–27·9 kg/m^2^; obesity, ≥ 28·0 kg/m^2^. The curves represent OR (solid lines) and 95 % CI (long dashed lines) for the effect of GWG at the end of the second trimester on composite adverse outcomes. The model was adjusted for maternal age, maternal height, gestational age of weight measure, parity (primipara or multipara), history of stillbirth (no verse yes), education (≤ 12 years or > 12 years) and active smoking (no verse yes). The reference values were set at the 50th percentiles (OR = 1), and the knots in the default positions were placed at the 5th, 35th, 65th and 95th percentiles of GWG. The histograms represent the distribution of GWG in our cohort, excluding values outside the −3 sd and +3 sd. GWG, gestational weight gain

Figure 1 shows the RCS curves for the relationship between *composite adverse outcomes* and GWG in prepregnancy BMI subgroups. The OR (95 % CI) for *composite adverse outcomes* changed smoothly across the GWG continuum, so there were no apparent cut-offs to identify recommended ranges of GWG. We apply the non-inferiority margin to select the reference values. The lowest point was very close to the median (P50) of GWG (Table 2), so we set P50 as the reference value (OR = 1)^(19)^. We tried to search the value that the 95 % CI closest within the 10 % non-inferiority margin (OR = 1·1) to establish the ranges of GWG^(18)^ (online Supplementary eTable 1).

**Table 2:** Values for establishing a recommended range of weight gain at the end of the second trimester

Prepregnancy	*Y* = composite adverse outcome				Noninferiority margins at 10 %					
BMI Group	OR = 1 (P50)	Nadir point	OR	95 % CI	Lower cut-off	OR	95 % CI	Upper cut-off	OR	95 % CI
	kg	kg			kg			kg		
Underweight	8·5	10·5	0·952	0·874, 1·037	7·8	1·046	1·002, 1·092	12·3	0·984	0·881, 1·099
Normal weight	8·0	7·5	0·998	0·988, 1·008	4·8	1·056	1·017, 1·096	10·0	1·061	1·027, 1·095
Overweight	7·0	3·2	0·948	0·892, 1·008	0·1	0·956	0·833, 1·098	8·6	1·057	1·017, 1·099
Obesity	5·5	4·5	0·998	0·962, 1·035	1·0	1·004	0·916, 1·099	7·3	1·030	0·969, 1·095

Prepregnancy BMI groups were divided following the Chinese standard: underweight, < 18·5 kg/m^2^; normal weight, 18·5–23·9 kg/m^2^; overweight, 24·0–27·9 kg/m^2^; obesity, ≥ 28·0 kg/m^2^. OR were estimated from a logistic regression model adjusted for maternal age, maternal height, gestational age of weight measure, parity (primipara or multipara), history of stillbirth (no verse yes), education (≤ 12 years or > 12 years) and active smoking (no verse yes).

In the underweight group (Fig. 1(a)), the gentle L-shaped curve with a warped tail highlights that the lower GWG was linked with increased risks of composite adverse outcomes (*P*-overall < 0·001, *P*-non-linear = 0·006). The nadir of risk occurred at a GWG of 10·5 kg. At the lower end of the L-shape, the 95 % CI first included the 10 % non-inferiority margin at a GWG at 7·8 kg (adjusted OR = 1·046, 95 % CI 1·002, 1·092) (Table 2). At the upper end of the curve, the 95 % CI remained within the 10 % margin until a GWG at 12·3 kg (adjusted OR = 0·984, 95 % CI 0·881, 1·099). The GWG between these two values represented that their risks of composite adverse outcomes were not meaningfully increased (< 10 %). In the normal weight group (Fig. 1(b)), we observed a U-shaped curve in which the nadir of risk occurred at a GWG of 7·5 kg (*P*-overall < 0·001, *P*-non-linear < 0·001). The recommended GWG was 4·8–10·0 kg (adjusted OR = 1·056, 95 % CI 1·017, 1·096; adjusted OR = 1·061, 95 % CI 1·027, 1·095) (Table 2). We repeated this process in the overweight and obesity groups, and Fig. 1(c) and 1(d) shows J-shape curves with a rise higher than the median value of GWG (7·0 kg, 5·5 kg). The 95 % CI first included the 10 % non-inferiority margin at a GWG at 0·1 kg and 1·0 kg (adjusted OR = 0·956, 95 % CI 0·833, 1·098; adjusted OR = 1·004, 95 % CI 0·916, 1·099). At the upper end of the curve, 95 % CI remained within the 10 % margin until a GWG at 8·6 kg and 7·3 kg (adjusted OR = 1·057, 95 % CI 1·017, 1·099; adjusted OR = 1·030, 95 % CI 0·969, 1·095) (Table 2).

### Gestational weight gain and risk score of adverse outcomes

**Fig. 2:**
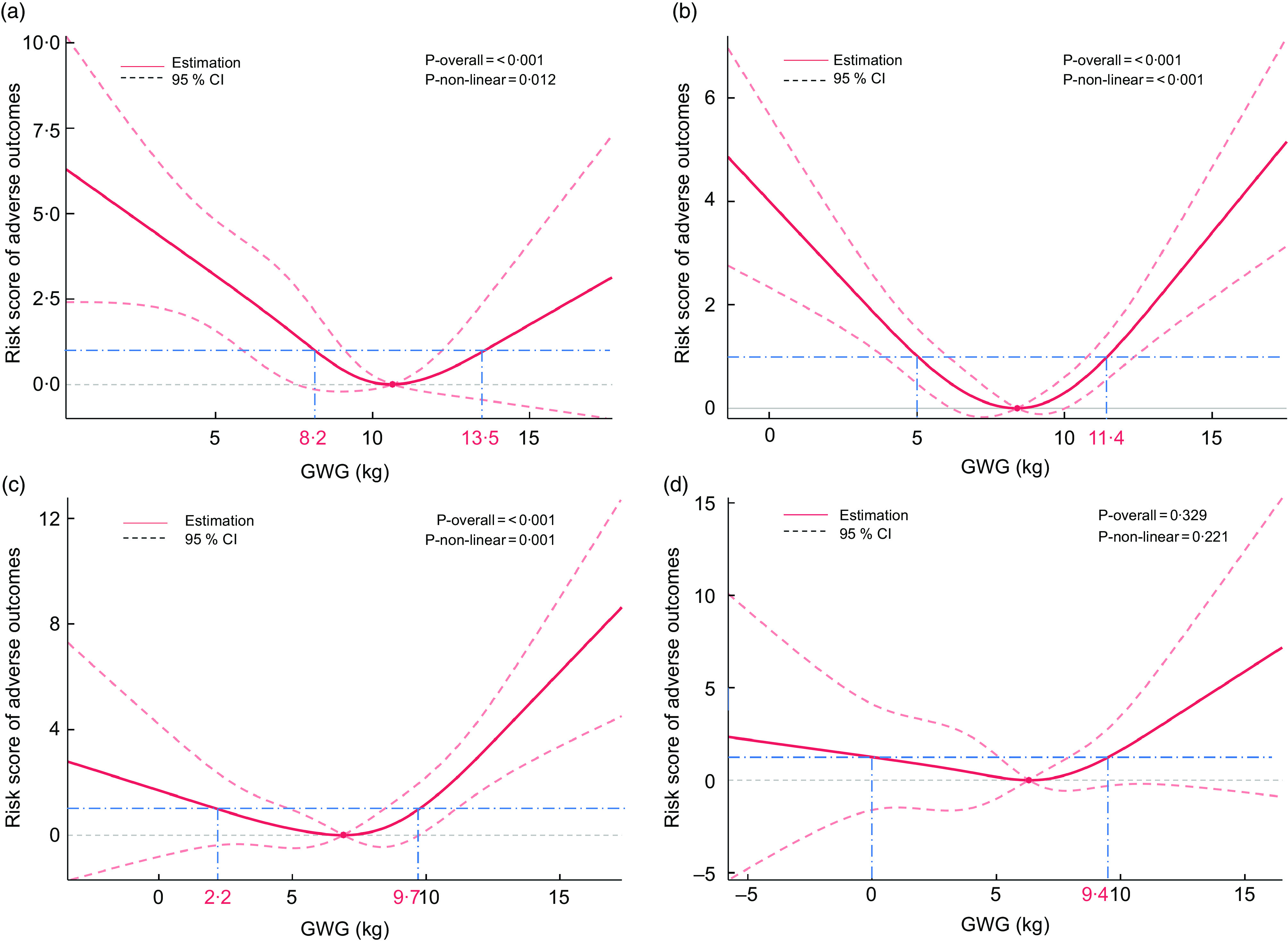
Restricted cubic spline for the associations between weight gain and risk score of adverse outcomes. (a) underweight group, (b) normal weight group, (c) overweight group, (d) obesity group. Prepregnancy BMI groups were divided following the Chinese standard: underweight, < 18·5 kg/m^2^; normal weight, 18·5–23·9 kg/m^2^; overweight, 24·0–27·9 kg/m^2^; obesity, ≥ 28·0 kg/m^2^. The curves represent the effect of GWG at the end of the second trimester on the risk score. The model was adjusted for maternal age, maternal height, gestational age of weight measure, parity (primipara or multipara), history of stillbirth (no verse yes), education (≤ 12 years or > 12 years) and active smoking (no verse yes). The reference values were set at the nadir of risk (*y* = 0). The knots in the default positions were placed at the 5th, 35th, 65th and 95th percentiles of the GWG. The solid lines represent estimation, the long dashed lines represent 95 % confidence intervals and the dashed dot lines represent the possible threshold (*y* = 1). Values outside the −3 sd and +3 sd were excluded. GWG, gestational weight gain

The risk score for pregnant women in the cohort ranged from 0 to 315. In the cohort, 48 683 women scored zero, meaning no adverse outcome. Besides them, the 25th, 50th and 75th percentiles of the risk scores were 30, 55 and 80. RCS examined possible non-linear relationships between GWG and risk score of adverse outcomes. The knots were selected according to the Akaike information criterion. As shown in Fig. 2, GWG and risk score were non-linearly correlated in the underweight, normal weight and overweight groups (*P*-overall < 0·05, *P*-non-linear < 0·05). However, it is not statistically significant in the obesity group (*P*-overall = 0·329, *P*-non-linear = 0·221). Based on the shape of the RCS, we found the GWG value at the nadir of the estimation curve was 10·6, 8·4, 6·9 and 6·3 kg in the underweight, normal weight, overweight and obesity groups, respectively. We take them as reference values (*y* = 0) and try to set boundaries at *y* = 1. The reasonable recommendation ranges of GWG may be 8·2–13·5 kg, 5·0–11·4 kg, 2·2–9·7 kg and 0–9·4 kg in these four groups because the risk score was only slightly increased (< 1 unit) within these boundaries.

After drawing up the lower and upper boundaries, we compared the risk scores of the appropriate (lower–upper cut-off), insufficient (< lower cut-off) and excessive (> upper cut-off) GWG categories (Table 3). The women with a recommended GWG have significantly lower risk scores than that of insufficient and excessive GWG in the underweight, normal weight and overweight groups (*P* < 0·05), but there was no statistical difference in the obesity group (*P* > 0·05).

**Table 3:** The cut-off of recommended weight gain at the end of the second trimester and comparison of the risk scores

				Mean (sem) of GWG							
Prepregnancy	GWG at *Y* = 0	GWG at *Y* = 1		Lower–higher cut-off		< lower cut-off		> upper cut-off			
BMI Group	The nadir point	Lower cut-off	Upper cut-off	Mean	sem	Mean	sem	Mean	sem	*F*	*P*
Underweight	10·6	8·2	13·5	10·38	0·42	12·49	0·47	11·23	1·24	5·776	0·003
Normal weight	8·4	5·0	11·4	13·85	0·17	15·88	0·39	15·47	0·38	17·425	< 0·001
Overweight	6·9	2·2	9·7	22·29	0·36	23·18	1·12	24·64	0·67	4·959	0·007
Obesity	6·3	0·0	9·4	35·27	0·67	36·87	2·22	36·69	1·50	0·552	0·576

Prepregnancy BMI groups were divided following the Chinese standard: underweight, < 18·5 kg/m^2^; normal weight, 18·5–23·9 kg/m^2^; overweight, 24·0–27·9 kg/m^2^; obesity, ≥ 28·0 kg/m^2^. Comparison between groups was adopted by one-way ANOVA.

### Extension evaluation of risk grade

We attempted to segment five risk grades based on the risk scores of adverse outcomes from low to high. It considered the frequency distribution of scores in the population (the risk scores 25th, 50th and 75th percentiles were 30, 55 and 80) and the severity of each outcome (online Supplementary eTable 2). A risk grade of 0 indicates no adverse outcome (*n* 48 683), 1 indicates LGA (*n* 7016), 2 indicates SGA or GDM (*n* 7909), 3 indicates HDP or preterm birth (2733) and 4 indicates stillbirth or two or more adverse outcomes (*n* 3518). We repeated the above method to fit the RCS curve, as the Y-axis represents the risk grades. The curve shape is similar to that of the risk score. The nadir was 11·4, 8·7, 7·4 and 5·4 kg, and the limits of candidates (*y* = 0·1) were > 6·7, 3·0–14·8, < 12·0 and −5·0–12·4 kg in the underweight, normal weight, overweight and obesity groups (online Supplementary eFig. 2).

## Discussion

The gestational period is an opportunity to improve positive health behaviours that can have both short- and long-term benefits for the mother and children. This study attempts to establish optimal ranges of GWG at the end of the second trimester to promote scientific weight management during pregnancy.

There are many challenges in establishing a recommended GWG. We must consider carefully which adverse events should be included to determine the optimal weight gain. Maternal and child health outcomes associated with GWG include HDP, GDM, preterm birth, SGA, LGA, fetal/infant death, cesarean delivery, postpartum weight retention, child obesity and longer term metabolic disorders in the mother^(3,5,6,14)^. First, considering multiple health outcomes is a dilemma because the relationship between individual adverse events and GWG is very different, even opposite (e.g. SGA and LGA). This study combined six adverse events (stillbirth, preterm birth, HDP, GDM, SGA and LGA) into a single *composite adverse outcome*. Here is something to explain: (1) cesarean delivery was not included mainly because the local cesarean delivery rate is as high as 51·1 % (Table 1). Planned or unplanned cesarean delivery was not identified separately in our healthcare system. Furthermore, the top three indications of cesarean delivery were scarred uterus, breech position, macrosomia (absolute indication); relative cephalopelvic disproportion, fetal distress in the uterus and social factor (relative indication) reported in the official report for 2015. Suppose cesarean delivery was included in the *composite adverse outcomes*. In that case, it might lead to an over high incidence and the incorporation of factors unrelated to GWG (such as the scarred uterus and social factors). (2) The mothers’ postpartum weight retention and metabolic diseases were related to GWG. However, they were also related to other factors, including diet, exercise, puerperal diseases and psychosocial status after childbirth. At the time, this information was not available. Therefore, we did not include the two in our evaluation system. (3) Moreover, we have already reported the association between GWG and childhood obesity according to the data on the growth and development of offspring from 0 to 6 years in the cohort^(20)^. The short- and long-term obesity of offspring is related to maternal weight, breast-feeding, dietary supplements and their family’s economic and educational level. In addition, childhood physical activity and post-milk diet are effective modifiers for childhood obesity. As a result, we have not included childhood obesity in the *composite adverse outcomes*. After considering the above reasons, we took parturition as the end of observation time and determined the inclusion of these six outcomes. Furthermore, it is worth thinking about how to integrate these health outcomes. It is imperative to evaluate the severity of each adverse health outcome scientifically. We calculated the risk score to weight six outcomes according to their severity rating based on a credible severity-weighting tool^(14)^. In this way, we regard multiple pregnancy outcomes as a whole instead of focusing on independent adverse events.

Notably, a systematic process to establish weight-gain thresholds was crucial^(18)^. Visually inspected curves to estimate the range of GWG was a common method. Nevertheless, it was subjective and may be either excessively narrow or wide. It may lead to unnecessary clinical intervention or failure to identify women benefitting from intervention to optimise weight gain. The non-inferiority test is a worthy candidate^(18)^. Non-inferiority margins can quantifiably identify points at which risks increase meaningfully. The margin is typically selected using expert clinical opinion or statistical limits, and a margin of 10 % was usually recommended^(18)^. In this study, the ranges provided in the result section may be one of many options. The supplementary document shows the OR (95 % CI) of adverse outcomes corresponding to different GWG (online Supplementary eTable 1). It provides more candidates for optimal ranges. Furthermore, the ranges we get based on the RCS from the risk scores were only one of the alternative boundaries. They were obtained after considering the width of the value ranges and the clinical significance.

The recommended values of GWG obtained by different outcome variables (composite adverse outcome, risk score and risk grade) were different (online Supplementary eTable 3). The value of nadir risk obtained by the weighted risk score is higher than that from the composite adverse outcome (with equal weight for each outcome). The main reason may be that preterm birth (related to less GWG) has greater weight than LGA (related to more GWG) in outcome variables, so the ranges of optimal GWG move upward. In addition, the evaluation method of risk grade has better clinical practicability than the risk score. Labelling pregnant women with different risk grades (non/low/medium/high/very high) can promote the identification and compliance with pregnancy weight management. In the study, the ranges of optimal GWG derived from risk grades were broader than that from risk scores. Generally, GWG at the end of the second trimester should not be too low for underweight pregnant women, preferably not < 7 kg. For most normal-weight women, a GWG of about 8 kg is optimal. Pregnant women who are overweight should not have a GWG of more than 9 kg and a maximum of 12 kg. The association between GWG and the risk of adverse outcomes was weak in women with obesity. That may be because obesity itself implies a high risk. However, we advised women with overweight and obesity to keep positive growth of GWG (> 0 kg) in the first and second trimesters (should better not be < 2·2 kg and 1·0 kg). Significant weight loss (> 5·0 kg), whether due to active weight control or hyperemesis gravida, may be detrimental to health. The exploration of recommended GWG in this study is devoted to providing a reference for prenatal care. It is not intended to replace expert opinion but to provide quantitative evidence to inform expert opinion better.

### Conclusions

According to the comprehensive adverse maternal and infant outcomes, we tried to recommend the optimal reference value and ranges of GWG at the end of the second trimester. For pregnant women with different BMI before pregnancy, individualised strategies should be adopted in weight management during pregnancy.

## Supplementary material

For supplementary material accompanying this paper visit https://doi.org/10.1017/S1368980023001490. click here to view supplementary material
