# Supplementary material for: Identifying optimal ranges of weight gain at the end of the second trimester result from a population-based cohort study
Source: Public Health Nutr. 2023 Aug 14;26(10):2005–13. doi: 10.1017/S1368980023001490 (PMC10564611; doi:10.1017/S1368980023001490)
Supplement: Supplementary file 1 [file S1368980023001490sup001.docx]

**Supplement file**


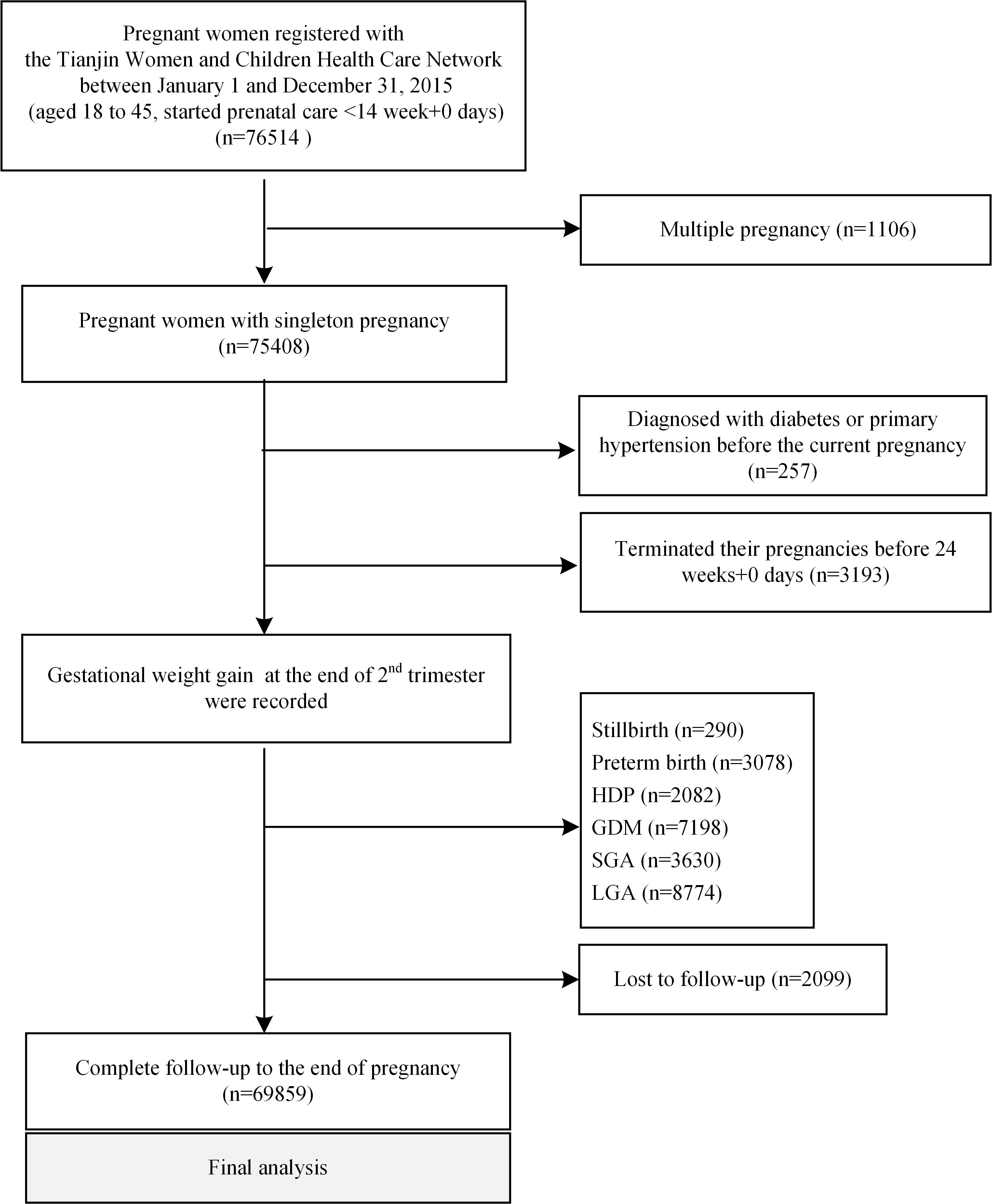


**eFigure 1.** Study flow chart

Abbreviations: HDP = hypertensive disorders of pregnancy, GDM = gestational diabetes mellitus, SGA = small for gestational age, LGA = large for gestational age.


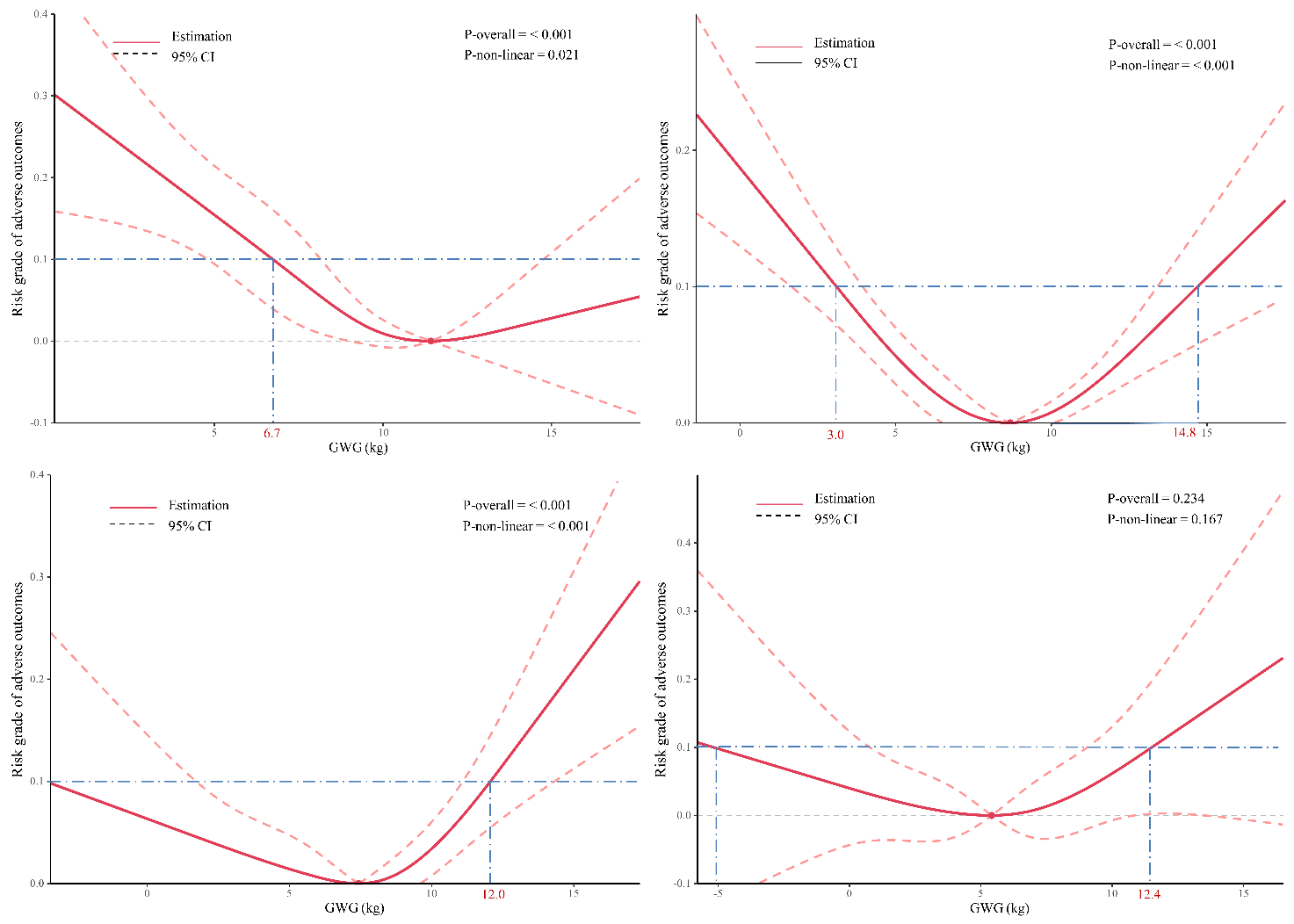


**eFigure 2.** Restricted cubic spline for the associations between weight gain and risk grade of adverse outcomes.

A. underweight group, B. normal weight group, C. overweight group, D. obesity group. Prepregnancy BMI groups were divided following the Chinese standard: underweight, <18.5 kg/m^2^; normal weight, 18.5–23.9 kg/m^2^; overweight, 24.0–27.9 kg/m^2^; obesity, ≥ 28.0 kg/m^2^. The curves represent the effect of GWG at the end of the second trimester on the risk grade. The model was adjusted for maternal age, maternal height, gestational age of weight measure, parity (primipara or multipara), history of stillbirth (no verse yes), education (≤12 years or >12 years), and active smoking (no verse yes). The reference values were set at the nadir of risk (y=0). The knots in the default positions were set at the 5th, 35th, 65th, and 95th percentiles of the GWG. The solid lines represent estimation, the long dashed lines represent 95% confidence intervals, and the dashed dot lines represent the possible threshold (y=0.1). Values outside the -3SD and +3SD were excluded. GWG = gestational weight gain.

**eTable 1.** Odds Ratios and 95% Confidence Intervals for the Effects of Weight Gain at the end of the second trimester on the Composite Adverse Outcome

| **Underweight Group** | |  | **Normal weight Group** | |
| --- | --- | --- | --- | --- |
| **GWG (kg)** | **OR (95% CI)** |  | **GWG (kg)** | **OR (95% CI)** |
| 7.0 | 1.103 (1.029 ~ 1.182) |  | 4.0 | 1.087 (1.039 ~ 1.138) |
| 7.1 | 1.096 (1.025 ~ 1.172) |  | 4.1 | 1.083 (1.036 ~ 1.132) |
| 7.2 | 1.089 (1.021 ~ 1.162) |  | 4.2 | 1.079 (1.034 ~ 1.126) |
| 7.2 | 1.083 (1.017 ~ 1.153) |  | 4.3 | 1.075 (1.031 ~ 1.121) |
| 7.3 | 1.076 (1.014 ~ 1.143) |  | 4.4 | 1.071 (1.028 ~ 1.116) |
| 7.4 | 1.070 (1.011 ~ 1.133) |  | 4.5 | 1.067 (1.026 ~ 1.110) |
| 7.5 | 1.064 (1.008 ~ 1.123) |  | 4.6 | 1.063 (1.023 ~ 1.105) |
| 7.6 | 1.058 (1.006 ~ 1.113) |  | 4.7 | 1.059 (1.020 ~ 1.100) |
| 7.7 | 1.052 (1.004 ~ 1.102) |  | ***4.8*** | ***1.056 (1.017 ~ 1.096)*** |
| ***7.8*** | ***1.046 (1.002 ~ 1.092)*** |  | 4.9 | 1.052 (1.015 ~ 1.091) |
| 7.8 | 1.040 (1.001 ~ 1.082) |  | 5.0 | 1.049 (1.012 ~ 1.087) |
| 7.9 | 1.035 (1.000 ~ 1.071) |  | 5.1 | 1.045 (1.009 ~ 1.083) |
| 8.0 | 1.029 (0.999 ~ 1.061) |  | 5.2 | 1.042 (1.007 ~ 1.078) |
| 8.1 | 1.024 (0.998 ~ 1.051) |  | 5.3 | 1.039 (1.004 ~ 1.075) |
| 8.2 | 1.019 (0.998 ~ 1.040) |  | 5.4 | 1.036 (1.002 ~ 1.071) |
| 8.3 | 1.014 (0.998 ~ 1.030) |  | 5.4 | 1.033 (0.999 ~ 1.067) |
| 8.4 | 1.009 (0.999 ~ 1.020) |  | 5.5 | 1.030 (0.997 ~ 1.064) |
| 8.5 | 1.005 (0.999 ~ 1.011) |  | 5.6 | 1.027 (0.995 ~ 1.060) |
| **8.5** | **1.000 (1.000 ~ 1.001)** |  | 5.7 | 1.024 (0.992 ~ 1.057) |
| 8.6 | 0.996 (0.992 ~ 1.001) |  | 5.8 | 1.022 (0.990 ~ 1.054) |
| 8.7 | 0.992 (0.982 ~ 1.002) |  | 5.9 | 1.019 (0.989 ~ 1.050) |
| 8.8 | 0.988 (0.974 ~ 1.003) |  | 6.0 | 1.017 (0.987 ~ 1.047) |
| 8.9 | 0.985 (0.965 ~ 1.005) |  | 6.1 | 1.014 (0.985 ~ 1.045) |
| 9.0 | 0.981 (0.957 ~ 1.006) |  | 6.2 | 1.012 (0.984 ~ 1.042) |
| 9.1 | 0.978 (0.949 ~ 1.008) |  | 6.3 | 1.010 (0.983 ~ 1.039) |
| 9.2 | 0.975 (0.941 ~ 1.009) |  | 6.4 | 1.009 (0.982 ~ 1.036) |
| 9.2 | 0.972 (0.934 ~ 1.011) |  | 6.5 | 1.007 (0.981 ~ 1.033) |
| 9.3 | 0.969 (0.927 ~ 1.013) |  | 6.6 | 1.005 (0.980 ~ 1.031) |
| 9.4 | 0.966 (0.920 ~ 1.015) |  | 6.7 | 1.004 (0.980 ~ 1.028) |
| 9.5 | 0.964 (0.914 ~ 1.017) |  | 6.8 | 1.003 (0.980 ~ 1.026) |
| 9.6 | 0.962 (0.908 ~ 1.019) |  | 6.9 | 1.001 (0.980 ~ 1.023) |
| 9.7 | 0.960 (0.903 ~ 1.020) |  | **7.0** | **1.000 (0.981 ~ 1.021)** |
| 9.8 | 0.958 (0.898 ~ 1.022) |  | 7.1 | 1.000 (0.981 ~ 1.018) |
| 9.9 | 0.957 (0.894 ~ 1.024) |  | 7.2 | 0.999 (0.982 ~ 1.016) |
| 9.9 | 0.955 (0.890 ~ 1.026) |  | 7.3 | 0.998 (0.983 ~ 1.014) |
| 10.0 | 0.954 (0.886 ~ 1.028) |  | 7.3 | 0.998 (0.985 ~ 1.012) |
| 10.1 | 0.953 (0.883 ~ 1.030) |  | 7.4 | 0.998 (0.986 ~ 1.010) |
| 10.2 | 0.953 (0.880 ~ 1.031) |  | **7.5** | **0.998 (0.988 ~ 1.008)** |
| 10.3 | 0.952 (0.878 ~ 1.033) |  | 7.6 | 0.998 (0.990 ~ 1.006) |
| 10.4 | 0.952 (0.876 ~ 1.035) |  | 7.7 | 0.998 (0.992 ~ 1.004) |
| **10.5** | **0.952 (0.874 ~ 1.037)** |  | 7.8 | 0.999 (0.995 ~ 1.003) |
| 10.6 | 0.952 (0.873 ~ 1.038) |  | 7.9 | 0.999 (0.998 ~ 1.001) |
| 10.6 | 0.952 (0.871 ~ 1.040) |  | **8.0** | **1.000 (1.000 ~ 1.000)** |
| 10.7 | 0.953 (0.871 ~ 1.042) |  | 8.1 | 1.001 (0.999 ~ 1.004) |
| 10.8 | 0.953 (0.870 ~ 1.044) |  | 8.2 | 1.002 (0.998 ~ 1.007) |
| 10.9 | 0.954 (0.870 ~ 1.046) |  | 8.3 | 1.004 (0.997 ~ 1.010) |
| 11.0 | 0.955 (0.869 ~ 1.048) |  | 8.4 | 1.005 (0.996 ~ 1.014) |
| 11.1 | 0.956 (0.869 ~ 1.051) |  | 8.5 | 1.007 (0.996 ~ 1.017) |
| 11.2 | 0.957 (0.870 ~ 1.053) |  | 8.6 | 1.009 (0.996 ~ 1.021) |
| 11.3 | 0.958 (0.870 ~ 1.055) |  | 8.7 | 1.011 (0.996 ~ 1.025) |
| 11.3 | 0.960 (0.870 ~ 1.058) |  | 8.8 | 1.013 (0.996 ~ 1.030) |
| 11.4 | 0.961 (0.871 ~ 1.061) |  | 8.9 | 1.015 (0.997 ~ 1.034) |
| 11.5 | 0.963 (0.872 ~ 1.064) |  | 9.0 | 1.018 (0.998 ~ 1.038) |
| 11.6 | 0.965 (0.873 ~ 1.067) |  | 9.1 | 1.021 (0.999 ~ 1.043) |
| 11.7 | 0.967 (0.874 ~ 1.070) |  | 9.2 | 1.024 (1.001 ~ 1.048) |
| 11.8 | 0.969 (0.875 ~ 1.073) |  | 9.3 | 1.027 (1.002 ~ 1.053) |
| 11.9 | 0.971 (0.876 ~ 1.077) |  | 9.3 | 1.031 (1.005 ~ 1.057) |
| 11.9 | 0.973 (0.877 ~ 1.081) |  | 9.4 | 1.034 (1.007 ~ 1.062) |
| 12.0 | 0.976 (0.878 ~ 1.085) |  | 9.5 | 1.038 (1.010 ~ 1.068) |
| 12.1 | 0.978 (0.879 ~ 1.090) |  | 9.6 | 1.042 (1.013 ~ 1.073) |
| 12.2 | 0.981 (0.880 ~ 1.094) |  | 9.7 | 1.047 (1.016 ~ 1.078) |
| ***12.3*** | ***0.984 (0.881 ~ 1.099)*** |  | 9.8 | 1.051 (1.019 ~ 1.084) |
| 12.4 | 0.987 (0.881 ~ 1.104) |  | 9.9 | 1.056 (1.023 ~ 1.089) |
| 12.5 | 0.989 (0.882 ~ 1.110) |  | ***10.0*** | ***1.061 (1.027 ~ 1.095)*** |
| 12.6 | 0.992 (0.883 ~ 1.115) |  | 10.1 | 1.066 (1.031 ~ 1.101) |
| 12.6 | 0.996 (0.884 ~ 1.122) |  | 10.2 | 1.071 (1.036 ~ 1.107) |
| 12.7 | 0.999 (0.884 ~ 1.128) |  | 10.3 | 1.076 (1.040 ~ 1.113) |
| 12.8 | 1.002 (0.885 ~ 1.134) |  | 10.4 | 1.082 (1.045 ~ 1.120) |
| 12.9 | 1.005 (0.885 ~ 1.141) |  | 10.5 | 1.088 (1.050 ~ 1.126) |
| 13.0 | 1.009 (0.886 ~ 1.149) |  | 10.6 | 1.094 (1.056 ~ 1.133) |

| **Overweight Group** | |  | **Obesity Group** | |
| --- | --- | --- | --- | --- |
| **GWG (kg)** | **OR (95% CI)** |  | **GWG (kg)** | **OR (95% CI)** |
| -0.3 | 0.958 (0.823 ~ 1.114) |  | -0.1 | 1.007 (0.894 ~ 1.133) |
| -0.2 | 0.957 (0.826 ~ 1.110) |  | 0.0 | 1.006 (0.897 ~ 1.129) |
| -0.1 | 0.957 (0.828 ~ 1.106) |  | 0.2 | 1.006 (0.900 ~ 1.125) |
| 0.0 | 0.957 (0.831 ~ 1.102) |  | 0.3 | 1.006 (0.902 ~ 1.121) |
| ***0.1*** | ***0.956 (0.833 ~ 1.098)*** |  | 0.4 | 1.005 (0.905 ~ 1.117) |
| 0.3 | 0.956 (0.835 ~ 1.094) |  | 0.5 | 1.005 (0.907 ~ 1.114) |
| 0.4 | 0.956 (0.838 ~ 1.090) |  | 0.6 | 1.005 (0.909 ~ 1.110) |
| 0.5 | 0.955 (0.840 ~ 1.086) |  | 0.7 | 1.004 (0.911 ~ 1.107) |
| 0.6 | 0.955 (0.843 ~ 1.082) |  | 0.8 | 1.004 (0.913 ~ 1.104) |
| 0.7 | 0.955 (0.845 ~ 1.078) |  | 0.9 | 1.004 (0.915 ~ 1.102) |
| 0.8 | 0.954 (0.848 ~ 1.074) |  | ***1.0*** | ***1.004 (0.916 ~ 1.099)*** |
| 0.9 | 0.954 (0.850 ~ 1.070) |  | 1.2 | 1.003 (0.918 ~ 1.097) |
| 1.0 | 0.954 (0.852 ~ 1.067) |  | 1.3 | 1.003 (0.919 ~ 1.094) |
| 1.1 | 0.953 (0.855 ~ 1.063) |  | 1.4 | 1.003 (0.920 ~ 1.092) |
| 1.2 | 0.953 (0.857 ~ 1.059) |  | 1.5 | 1.002 (0.922 ~ 1.090) |
| 1.3 | 0.953 (0.859 ~ 1.056) |  | 1.6 | 1.002 (0.923 ~ 1.089) |
| 1.4 | 0.952 (0.862 ~ 1.052) |  | 1.7 | 1.002 (0.924 ~ 1.087) |
| 1.5 | 0.952 (0.864 ~ 1.049) |  | 1.8 | 1.002 (0.924 ~ 1.085) |
| 1.6 | 0.952 (0.866 ~ 1.045) |  | 1.9 | 1.001 (0.925 ~ 1.084) |
| 1.7 | 0.951 (0.868 ~ 1.042) |  | 2.1 | 1.001 (0.926 ~ 1.082) |
| 1.8 | 0.951 (0.870 ~ 1.039) |  | 2.2 | 1.001 (0.927 ~ 1.081) |
| 1.9 | 0.951 (0.872 ~ 1.036) |  | 2.3 | 1.001 (0.927 ~ 1.080) |
| 2.0 | 0.950 (0.874 ~ 1.033) |  | 2.4 | 1.000 (0.928 ~ 1.079) |
| 2.1 | 0.950 (0.876 ~ 1.030) |  | 2.5 | 1.000 (0.929 ~ 1.077) |
| 2.2 | 0.950 (0.878 ~ 1.027) |  | **2.6** | **1.000 (0.930 ~ 1.076)** |
| 2.3 | 0.949 (0.880 ~ 1.024) |  | 2.7 | 1.000 (0.930 ~ 1.075) |
| 2.4 | 0.949 (0.882 ~ 1.022) |  | 2.8 | 1.000 (0.931 ~ 1.073) |
| 2.5 | 0.949 (0.884 ~ 1.019) |  | 3.0 | 0.999 (0.932 ~ 1.072) |
| 2.6 | 0.949 (0.885 ~ 1.017) |  | 3.1 | 0.999 (0.933 ~ 1.070) |
| 2.7 | 0.949 (0.887 ~ 1.015) |  | 3.2 | 0.999 (0.934 ~ 1.068) |
| 2.9 | 0.949 (0.888 ~ 1.013) |  | 3.3 | 0.999 (0.936 ~ 1.066) |
| 3.0 | 0.948 (0.890 ~ 1.011) |  | 3.4 | 0.999 (0.937 ~ 1.065) |
| 3.1 | 0.948 (0.891 ~ 1.009) |  | 3.5 | 0.999 (0.939 ~ 1.062) |
| **3.2** | **0.948 (0.892 ~ 1.008)** |  | 3.6 | 0.999 (0.941 ~ 1.060) |
| 3.3 | 0.948 (0.894 ~ 1.007) |  | 3.7 | 0.998 (0.942 ~ 1.058) |
| 3.4 | 0.949 (0.895 ~ 1.005) |  | 3.8 | 0.998 (0.945 ~ 1.055) |
| 3.5 | 0.949 (0.896 ~ 1.004) |  | 4.0 | 0.998 (0.947 ~ 1.052) |
| 3.6 | 0.949 (0.897 ~ 1.003) |  | 4.1 | 0.998 (0.950 ~ 1.049) |
| 3.7 | 0.949 (0.898 ~ 1.003) |  | 4.2 | 0.998 (0.952 ~ 1.046) |
| 3.8 | 0.949 (0.899 ~ 1.002) |  | 4.3 | 0.998 (0.956 ~ 1.042) |
| 3.9 | 0.950 (0.900 ~ 1.002) |  | 4.4 | 0.998 (0.959 ~ 1.038) |
| 4.0 | 0.950 (0.902 ~ 1.001) |  | **4.5** | **0.998 (0.962 ~ 1.035)** |
| 4.1 | 0.951 (0.903 ~ 1.001) |  | 4.6 | 0.998 (0.966 ~ 1.031) |
| 4.2 | 0.951 (0.904 ~ 1.001) |  | 4.7 | 0.998 (0.970 ~ 1.027) |
| 4.3 | 0.952 (0.905 ~ 1.001) |  | 4.9 | 0.998 (0.974 ~ 1.022) |
| 4.4 | 0.952 (0.906 ~ 1.001) |  | 5.0 | 0.998 (0.979 ~ 1.018) |
| 4.5 | 0.953 (0.908 ~ 1.001) |  | 5.1 | 0.998 (0.983 ~ 1.014) |
| 4.6 | 0.954 (0.909 ~ 1.001) |  | 5.2 | 0.999 (0.988 ~ 1.010) |
| 4.7 | 0.955 (0.911 ~ 1.001) |  | 5.3 | 0.999 (0.993 ~ 1.006) |
| 4.8 | 0.956 (0.913 ~ 1.001) |  | 5.4 | 1.000 (0.998 ~ 1.002) |
| 4.9 | 0.957 (0.914 ~ 1.001) |  | **5.5** | **1.000 (0.998 ~ 1.003)** |
| 5.0 | 0.958 (0.916 ~ 1.001) |  | 5.6 | 1.001 (0.994 ~ 1.008) |
| 5.1 | 0.959 (0.919 ~ 1.001) |  | 5.7 | 1.002 (0.990 ~ 1.013) |
| 5.2 | 0.960 (0.921 ~ 1.002) |  | 5.9 | 1.003 (0.987 ~ 1.019) |
| 5.4 | 0.962 (0.924 ~ 1.002) |  | 6.0 | 1.004 (0.984 ~ 1.025) |
| 5.5 | 0.963 (0.927 ~ 1.002) |  | 6.1 | 1.005 (0.981 ~ 1.030) |
| 5.6 | 0.965 (0.930 ~ 1.002) |  | 6.2 | 1.007 (0.978 ~ 1.036) |
| 5.7 | 0.967 (0.933 ~ 1.002) |  | 6.3 | 1.008 (0.975 ~ 1.042) |
| 5.8 | 0.969 (0.937 ~ 1.002) |  | 6.4 | 1.010 (0.973 ~ 1.048) |
| 5.9 | 0.971 (0.940 ~ 1.002) |  | 6.5 | 1.012 (0.971 ~ 1.053) |
| 6.0 | 0.973 (0.944 ~ 1.002) |  | 6.6 | 1.014 (0.970 ~ 1.059) |
| 6.1 | 0.975 (0.949 ~ 1.001) |  | 6.8 | 1.016 (0.969 ~ 1.065) |
| 6.2 | 0.977 (0.953 ~ 1.001) |  | 6.9 | 1.018 (0.968 ~ 1.071) |
| 6.3 | 0.979 (0.958 ~ 1.001) |  | 7.0 | 1.021 (0.968 ~ 1.077) |
| 6.4 | 0.982 (0.963 ~ 1.001) |  | 7.1 | 1.024 (0.968 ~ 1.083) |
| 6.5 | 0.984 (0.969 ~ 1.001) |  | 7.2 | 1.027 (0.968 ~ 1.089) |
| 6.6 | 0.987 (0.974 ~ 1.000) |  | ***7.3*** | ***1.030 (0.969 ~ 1.095)*** |
| 6.7 | 0.990 (0.980 ~ 1.000) |  | 7.4 | 1.033 (0.970 ~ 1.101) |
| 6.8 | 0.993 (0.986 ~ 1.000) |  | 7.5 | 1.037 (0.972 ~ 1.107) |
| 6.9 | 0.996 (0.992 ~ 1.000) |  | 7.7 | 1.041 (0.974 ~ 1.113) |
| **7.0** | **0.999 (0.998 ~ 1.000)** |  | 7.8 | 1.045 (0.976 ~ 1.119) |
| 7.1 | 1.002 (1.000 ~ 1.004) |  | 7.9 | 1.049 (0.978 ~ 1.125) |
| 7.2 | 1.006 (1.000 ~ 1.011) |  | 8.0 | 1.054 (0.981 ~ 1.131) |
| 7.3 | 1.009 (1.001 ~ 1.017) |  | 8.1 | 1.058 (0.984 ~ 1.138) |
| 7.4 | 1.012 (1.001 ~ 1.024) |  | 8.2 | 1.063 (0.987 ~ 1.144) |
| 7.5 | 1.016 (1.001 ~ 1.031) |  | 8.3 | 1.068 (0.991 ~ 1.151) |
| 7.6 | 1.020 (1.002 ~ 1.038) |  | 8.4 | 1.073 (0.995 ~ 1.157) |
| 7.7 | 1.024 (1.003 ~ 1.045) |  | 8.5 | 1.078 (0.998 ~ 1.164) |
| 7.8 | 1.027 (1.004 ~ 1.052) |  | 8.7 | 1.084 (1.002 ~ 1.171) |
| 8.0 | 1.031 (1.005 ~ 1.058) |  | 8.8 | 1.089 (1.007 ~ 1.178) |
| 8.1 | 1.036 (1.007 ~ 1.065) |  | 8.9 | 1.095 (1.011 ~ 1.186) |
| 8.2 | 1.040 (1.008 ~ 1.072) |  | 9.0 | 1.101 (1.015 ~ 1.194) |
| 8.3 | 1.044 (1.010 ~ 1.079) |  | 9.1 | 1.107 (1.020 ~ 1.202) |
| 8.4 | 1.048 (1.012 ~ 1.086) |  | 9.2 | 1.113 (1.024 ~ 1.210) |
| 8.5 | 1.053 (1.014 ~ 1.093) |  | 9.3 | 1.119 (1.029 ~ 1.218) |
| ***8.6*** | ***1.057 (1.017 ~ 1.099)*** |  | 9.4 | 1.126 (1.033 ~ 1.227) |
| 8.7 | 1.062 (1.019 ~ 1.107) |  | 9.6 | 1.133 (1.038 ~ 1.236) |
| 8.8 | 1.067 (1.022 ~ 1.113) |  | 9.7 | 1.139 (1.042 ~ 1.246) |
| 8.9 | 1.072 (1.026 ~ 1.120) |  | 9.8 | 1.146 (1.047 ~ 1.255) |
| 9.0 | 1.077 (1.029 ~ 1.127) |  | 9.9 | 1.154 (1.051 ~ 1.266) |

**eTable 2.** Risk grade of adverse outcomes

| **Risk grade** | **Score range** | **the corresponding adverse outcomes** |
| --- | --- | --- |
| 1. No risks | 0 | none |
| 1. Low risk | 1~30 | LGA |
| 1. Medium risk | 31~55 | SGA or GDM |
| 1. High risk | 56~80 | HDP or preterm birth |
| 1. Very high risk | 81~ | Stillbirth; 2 or more adverse outcomes |

Abbreviations: HDP = hypertensive disorders of pregnancy, GDM = gestational diabetes mellitus, SGA = small for gestational age, LGA = large for gestational age.

**eTable 3.** Values for establishing recommended ranges of weight gain at the end of the second trimester

| **Prepregnancy BMI Group** | **Y= composite adverse outcome** | | |  | **Y= risk score** | | |  | **Y= risk grade** | | |
| --- | --- | --- | --- | --- | --- | --- | --- | --- | --- | --- | --- |
|  | **the nadir point** | lower cutoff | upper cutoff |  | **the nadir point** | lower cutoff | upper cutoff |  | **the nadir point** | lower cutoff | upper cutoff |
| Underweight | **10.5** | 7.8 | 12.3 |  | **10.6** | 8.2 | 13.5 |  | **11.4** | 6.7 | N/A |
| Normal weight | **7.5** | 4.8 | 10.0 |  | **8.4** | 5.0 | 11.4 |  | **8.7** | 3.0 | 14.8 |
| Overweight | **3.2** | 0.1 | 8.5 |  | **6.9** | 2.2 | 9.7 |  | **7.4** | N/A | 12 |
| Obesity | **4.5** | 1.0 | 7.3 |  | **6.3** | 0 | 9.4 |  | **5.4** | -5.0 | 12.4 |

Notes: Prepregnancy BMI groups were divided following the Chinese standard: underweight, <18.5 kg/m^2^; normal weight, 18.5–23.9 kg/m^2^; overweight, 24.0–27.9 kg/m^2^; obesity, ≥ 28.0 kg/m^2^.
